# Improving response inhibition systems in frontotemporal dementia with citalopram

**DOI:** 10.1093/brain/awv133

**Published:** 2015-05-22

**Authors:** Laura E. Hughes, Timothy Rittman, Ralf Regenthal, Trevor W. Robbins, James B. Rowe

**Affiliations:** 1 Department of Clinical Neurosciences, University of Cambridge, UK; 2 Medical Research Council Cognition and Brain Sciences Unit, Cambridge, UK; 3 Division of Clinical Pharmacology, Department of Pharmacology and Toxicology, University of Leipzig, Germany; 4 Department of Psychology, University of Cambridge, Cambridge, UK; 5 Behavioural and Clinical Neuroscience Institute, Cambridge, UK

**Keywords:** frontotemporal dementia, citalopram, magnetoencephalography, response inhibition, voxel-based morphometry

## Abstract

Disinhibition is a cardinal feature of the behavioural variant of frontotemporal dementia, presenting as impulsive and impetuous behaviours that are often difficult to manage. The options for symptomatic treatments are limited, but a potential target for therapy is the restoration of serotonergic function, which is both deficient in behavioural variant frontotemporal dementia and closely associated with inhibitory control. Based on preclinical studies and psychopharmacological interventions in other disorders, we predicted that inhibition would be associated with the right inferior frontal gyrus and dependent on serotonin. Using magnetoencephalography and electroencephalography of a Go-NoGo paradigm, we investigated the neural basis of behavioural disinhibition in behavioural variant frontotemporal dementia and the effect of selective serotonin reuptake inhibition on the neural systems for response inhibition. In a randomized double-blinded placebo-controlled crossover design study, 12 patients received either a single 30 mg dose of citalopram or placebo. Twenty age-matched healthy controls underwent the same magnetoencephalography/electroencephalography protocol on one session without citalopram, providing normative data for this task. In the control group, successful NoGo trials evoked two established indices of successful response inhibition: the NoGo-N2 and NoGo-P3. Both of these components were significantly attenuated by behavioural variant frontotemporal dementia. Cortical sources associated with successful inhibition in control subjects were identified in the right inferior frontal gyrus and anterior temporal lobe, which have been strongly associated with behavioural inhibition in imaging and lesion studies. These sources were impaired by behavioural variant frontotemporal dementia. Critically, citalopram enhanced the NoGo-P3 signal in patients, relative to placebo treatment, and increased the evoked response in the right inferior frontal gyrus. Voxel-based morphometry confirmed significant atrophy of inferior frontal gyrus, alongside insular, orbitofrontal and temporal cortex in our patient cohort. Together, these data suggest that the dysfunctional prefrontal cortical systems underlying response inhibition deficits in behavioural variant frontotemporal dementia can be partially restored by increasing serotonergic neurotransmission. The results support a translational neuroscience approach to impulsive neurological disorders and indicate the potential for symptomatic treatment of behavioural variant frontotemporal dementia including serotonergic strategies to improve disinhibition.

## Abstract (precis)

Disinhibition is a cardinal feature of behavioural variant frontotemporal dementia, arising from both frontal atrophy and serotonin depletion. Hughes *et al.* show that neurophysiological signatures of inhibition are reduced in frontotemporal dementia, and that citalopram rescues prefrontal neurophysiological deficits relative to placebo. Boosting serotoninergic transmission may facilitate management of disinhibition.

## Graphical abstract

**Figure:**
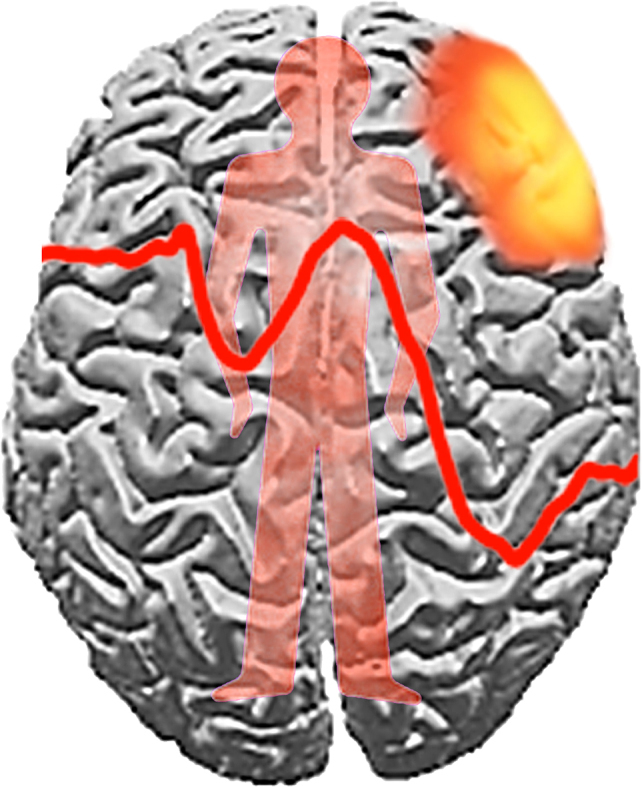
Disinhibition is a cardinal feature of behavioural variant frontotemporal dementia, arising from both frontal atrophy and serotonin depletion. Hughes *et al.* show that neurophysiological signatures of inhibition are reduced in frontotemporal dementia, and that citalopram rescues prefrontal neurophysiological deficits relative to placebo. Boosting serotoninergic transmission may facilitate management of disinhibition.

## Introduction

Behavioural deficits are a common and challenging aspect of the behavioural variant of frontotemporal dementia (bvFTD). Disinhibition, impulsivity and socially inappropriate behaviour are core diagnostic features of this disorder, together with stereotypical or perseverative actions, hyperorality, loss of empathy, apathy, and executive dysfunction including cognitive inflexibility (Rascovsky *et al.*, 2011). To advance new treatments for disinhibition and impulsivity in bvFTD, three requirements need to be addressed: first, is to understand the neural systems of these symptoms, drawing on comparative studies and insights from healthy and patient groups. Second, is to identify pharmacological targets for treatment based on the psychopharmacology of response inhibition and the neurotransmitter deficits in bvFTD. Third, is to have a sensitive marker to measure changes in the function of the neural systems underlying behavioural disinhibition.

Impulsivity in neurological and neuropsychiatric disorders is multifaceted, including premature and ill-considered actions, impaired behavioural restraint and suboptimal judgements of risk and outcome that together can result in adverse consequences. These different aspects of disinhibition are likely to arise from deficits in multiple cognitive and emotional processes, associated with different structural and neurochemical abnormalities. Here, we focus on one aspect of impulsivity, the deficit in response inhibition. The ability to inhibit an action is pivotal for self-control enabling flexible regulation of behaviour to withhold planned or habitual actions until an appropriate context or time. To examine response inhibition in bvFTD we adopted a Go-NoGo paradigm in which ‘Go’ trials induce a prepotent motor response that must be withheld on ‘NoGo’ trials. This type of paradigm is included as part of the frontal assessment battery, a sensitive measure of frontal impairment: patients with bvFTD are typically impaired at this task, failing to inhibit responses and making many commission errors (Dubois *et al.*, 2000; Slachevsky *et al.*, 2004). However, performance can be variable and patients with mild dementia are not always worse than controls (Collette *et al.*, 2007), suggesting that this task may be useful to investigate the spectrum of disease severity in bvFTD.

In health, NoGo inhibition recruits a broad cortical–subcortical network including lateral prefrontal cortices, anterior cingulate, premotor regions and subthalamic nucleus (Rubia *et al.*, 2001; Zheng *et al.*, 2008; Swick *et al.*, 2011). Within this network, the right inferior frontal gyrus has been identified as critical for inhibitory control (Konishi *et al.*, 1998, 1999; Aron *et al.*, 2004, 2014; Macoveanu *et al.*, 2013) and inhibitory failures are more common when this region is impaired after a lesion (Aron *et al.*, 2003; Kopp *et al.*, 2013) or after transcranial magnetic stimulation (Chambers *et al.*, 2006). Comparably, impulsive and disinhibited behaviours in bvFTD arise in part from focal atrophy of frontal cortex (Williams *et al.*, 2005; Seeley *et al.*, 2008; Kipps *et al.*, 2009; Pereira *et al.*, 2009; Whitwell *et al.*, 2009; Gordon *et al.*, 2010; Rohrer *et al.*, 2010) together with abnormal frontolimbic (Farb *et al.*, 2013) and frontostriatal connectivity (O'Callaghan *et al.*, 2013*a*, *b*).

Successful response inhibition is also dependent on serotonin (*cf*. Eagle *et al.*, 2008). For example, in humans and animals, serotonergic agonists enhance prefrontal responses during inhibition (Anderson *et al.*, 2002; Del-Ben *et al.*, 2005; Vollm *et al.*, 2006), whilst depleting serotonin impairs the ability to withhold a response (Harrison *et al.*, 1999; Eagle *et al.*, 2009) and reduces prefrontal activation (Rubia *et al.*, 2005). Studies of individual differences, such as genetic polymorphisms of the serotonin transporter or trait serotonin receptor density in the normal population, provide further evidence for the importance of serotonin within the neural systems for response inhibition. For example, NoGo paradigms have revealed that in healthy subjects a susceptibility to impulsivity is due in part to genetic variation in serotonergic systems and trait serotonergic function (Nomura and Nomura, 2006; Beste *et al.*, 2011; Macoveanu *et al.*, 2013). Patients with bvFTD have an altered cerebral neurochemical profile (Huey *et al.*, 2006), including severe reductions in the serotonergic innervation of the forebrain (Yang and Schmitt, 2001), reduced 5HT1a and 5HT2a receptors (Procter *et al.*, 1999; Bowen *et al.*, 2008), and altered serotonergic receptor binding in the frontal and temporal cortex (Sparks and Markesbery, 1991; Franceschi *et al.*, 2005; Lanctot *et al.*, 2007).

We propose that serotonergic deficiency and prefrontal dysfunction are directly relevant to behavioural disinhibition in bvFTD, and provide a compelling target for treatment. Selective serotonin reuptake inhibitors (SSRIs) are an attractive candidate therapy in this setting, given the well-established profile of safety and side-effects in adults. Acutely, they increase the extracellular levels of serotonin in the prefrontal cortex ∼4-fold (Bymaster *et al.*, 2002). SSRIs have been tested in a small number of open-label studies of bvFTD (for review see Boxer and Boeve, 2007; Freedman, 2007) with promising results, such as improvement on the Neuropsychiatric Inventory (Swartz *et al.*, 1997; Lebert *et al.*, 2004; Herrmann *et al.*, 2012;) and reduced stereotypical behaviours (Ikeda *et al.*, 2004; Mendez *et al.*, 2005; Ishikawa *et al.*, 2006). However, not all SSRIs reveal consistent improvements, with paroxetine for example leading to mixed results (Moretti *et al.*, 2003; Deakin *et al.*, 2004). Randomized placebo controlled studies are required to further establish efficacy but such clinical studies would benefit from evidence of the engagement of residual inhibitory systems in bvFTD, in addition to the preclinical evidence.

The potential effect of serotonergic therapies on disinhibition is distinct from the common clinical use for affective disorders. A benefit in treating impulsivity has been shown in the context of another neurological disorder associated with milder impulsivity and moderate serotonin deficiency, namely Parkinson’s disease (Ye *et al.*, 2014), despite clear differences in other aspects of the clinical syndrome. Because the slowing or reversal of neuropathology and neuronal loss in bvFTD is not at present possible, pharmacological manipulations that enhance serotonergic neurotransmitter function offer a potential means of restoring the function of brain networks underlying response inhibition.

To understand how serotonergic therapy with an SSRI affects inhibitory systems, we studied the neurophysiological signatures of successful NoGo trials using simultaneous magnetoencephalography (MEG) and electroencephalography (EEG) in a double-blind crossover study of citalopram. MEG and EEG are well tolerated by patients with bvFTD and are sensitive to the impact of bvFTD on local brain function and cortical networks (Hughes *et al.*, 2011, 2013; Hughes and Rowe, 2013). Electrophysiological studies have consistently identified two main components in the event-related potential (ERP) that index successful inhibition: the NoGo-N2 (Falkenstein *et al.*, 2002; Nieuwenhuis *et al.*, 2003; Falkenstein, 2006; Huster *et al.*, 2013, 2014; Smith *et al.*, 2013) and the NoGo-P3 (Band and van Boxtel, 1999; Roche *et al.*, 2005; Smith *et al.*, 2008, 2013; Enriquez-Geppert *et al.*, 2010). While EEG represents spatial summation of conducted neural activity, and is well suited to identifying the time course of significant components, MEG has the potential advantage in localizing cortical sources. Inferior frontal and temporal activity has been identified using MEG in association with NoGo inhibition in healthy adults (Vidal *et al.*, 2012) and adolescents (Vara *et al.*, 2014). Moreover, electrophysiological measures are sensitive to degenerative disease, with attenuated responses during NoGo in inhibition in Parkinson’s disease (Bokura *et al.*, 2005) and Huntington’s disease (Beste *et al.*, 2008).

Our principal hypothesis was that citalopram would restore the function of the deficient systems for successful inhibition in bvFTD, centred on the prefrontal cortex. The advantage of MEG with EEG over simple behavioural measures is not only to provide greater sensitivity to psychopharmacological intervention, but also to enable cross-validation of translational models of response inhibition systems. The latter may be achieved, for example, by examining serotonergic modulation of frontostriatal systems of inhibitory control in rats and non-human primates (cf. Robbins, 2007; Cools *et al.*, 2008). Such translational cross-validation may thus facilitate the use of response inhibition measures within clinical trials, especially where they are also correlated with neuropsychological and ecological measures of impulsivity.

We predicted that bvFTD would diminish the N2 and P3 components of the neurophysiological signature of response inhibition, with reduced neuronal source currents especially in the right inferior frontal and temporal regions. We also predicted that citalopram would restore the function of these frontal sources, in the context of serotonergic depletion caused by bvFTD.

## Materials and methods

### Subjects

Twelve right-handed adult patients with progressive bvFTD were recruited from the specialist FTD clinic at the Cambridge University Hospitals NHS Trust, based on clinical diagnostic criteria, including abnormal clinical imaging (Rascovsky *et al.*, 2011). Patients with non-progressive mimics of bvFTD were not included (Kipps *et al.*, 2010). Subjects were excluded if they were prescribed serotonergic reuptake inhibitors or if they had any contraindications to MEG or citalopram. A screening electrocardiogram was performed if a risk of cardiac disease was suggested by personal medical history (e.g. hypertension) or family history (e.g. cardiac disease). Twenty right-handed healthy older adults were recruited from the volunteer panel of the MRC Cognition and Brain Sciences Unit. No subjects in the control group had a history of significant neurological or psychiatric illness, nor reported any cognitive symptoms. The study was approved by the local Research Ethics Committee and exempted from Clinical Trials status by the United Kingdom’s Medicines and Healthcare products Regulatory Authority. All participants gave written informed consent before participation according to the 1991 Declaration of Helsinki.

Patients underwent neuropsychological assessment including the revised Addenbrooke’s Cognitive Examination (ACE-r) (Mioshi *et al.*, 2006), Mini-Mental State Examination (MMSE), the Hayling and Brixton Task (Burgess and Shallice, 1997), the Graded Naming test and the revised Beck Depression Inventory (BDI-II). On each session the Kirby Temporal Discounting Test and a Visual Analogue Scale were used to assess impulsivity and emotional state, respectively. Caregivers completed the Cambridge Behavioural Inventory (CBI) (Wedderburn *et al.*, 2008) to provide an assessment of the severity of behavioural symptoms. Patient and control details are summarized in Table 1.

**Table 1:** Details of patients with bvFTD and healthy control subjects

	Controls	Patients
*Male/Female*	8 m / 12f	6 m / 6f
*Age*	61.3 (9.32)	62.4 (6.0)
*MMSE*	29.6 (0.68)	24.1 (3.6)
*ACE-r*		
Total (100)	97.5 (1.99)	67.4 (16.5)
Attention (18)	17.75 (0.55)	14.9 (2.7)
Memory (26)	25.35 (1.04)	15.7 (7.1)
Verbal fluency (14)	12.9 (1.33)	4.2 (3.0)
Language (26)	25.7 (0.55)	18.8 (6.6)
Visual Spatial (16)	15.7 (0.55)	13.7 (1.6)
*Graded Naming*		11.7 (8.9)
*CBI*		
Total		116.7 (21.3)
Stereotypic and motor behaviours		14.2 (2.8)
Disinhibited phenotype scale*		40.4 (5.6)
*Hayling*		
A+B Errors**		37.6 (27.5)

Values shown are group means [standard deviation (SD) in parentheses]. MMSE = 30 point Mini-Mental State Examination; ACE-r = Addenbrooke’s cognitive exam revised, scored out of 100, divided into five subscales with total points for each in parentheses. The Hayling score is the converted error score on section two ‘unconnected completion’ (out of a possible 128). Graded naming is number of errors out of 30; CBI = Cambridge Behavioural Inventory.

*Composite sum from Cambridge Behavioural Inventory subscales including all items from the disinhibited, challenging, motor, eating and insight subscales, and the euphoria items from the mood subscale (Borroni *et al.*, 2012).

**For comparison, O'Callaghan *et al.*, (2013*b*) reported bvFTD patients A+B errors as 37.5 (19.7) and control A+B errors 1.4 (2.2).

### Experimental design

The bvFTD group were entered into a double-blind randomized crossover design. Two sessions were conducted ∼2 weeks apart, in which patients were given either 3 × 10 mg oral tablets of citalopram or 3 × 10 mg of placebo tablets. Blood samples were taken 2 h after drug administration, immediately before the MEG recording, close to the estimated time of peak plasma concentration (Sangkuhl *et al.*, 2011). The relationship between peak plasma and peak CNS levels is complex, but based on animal studies (Cremers *et al.*, 2009; Karlsson *et al.*, 2013) and observed midbrain SERT occupancy in healthy human volunteers (Klein *et al.*, 2006), we expected peak CNS levels over this timeframe. Mean plasma levels after citalopram, measured by a specific validated high performance chromatographic method, were 38.6 ng/ml (range 23.8–55.7 ng/ml) and after placebo, 0 ng/ml. Two patients did not complete the citalopram session, one because of nausea and one because of unexplained refusal, leaving 10 patients who completed both sessions. Control subjects were assessed on one occasion, for normative data and comparison with patients on placebo. Thus for the behavioural analyses, disease effects (versus controls) were examined from the 12 patients who completed the placebo session compared with the 20 controls, and serotonergic effects (citalopram versus placebo) were examined in the 10 patients who completed both sessions. The MEG and EEG analysis (described below) included only successful Go and NoGo trials. For the MEG/EEG results, two patients did not have enough successful NoGo trials to be included, leaving 10 patients on placebo to compare with controls and nine patients for the repeated measures design.

### Task

The Go-NoGo task comprised 400 visually cued Go trials and 104 visually cued NoGo trials, split into four equal blocks. Presentation of stimuli was controlled using EPrime®. Each trial started with a white fixation cross presented centrally on a dark grey background for 2 s followed by the presentation of a letter cue that subtended 0.8°. Go trials were cued with the letter ‘O’, presented centrally until the response button was pressed, or until 1.5 s if no press was made. NoGo trials were cued with the letter ‘X’ and were presented for 1.5 s. Stimulus onset asynchrony was 3.5 s. Trial order was pseudorandom, permuted such that on 15% of trials a NoGo cue was presented after each of 1, 3, 5, and 7 Go trials, and on 5% of trials the NoGo cue was presented after each of 0, 2, 4, 6, and 8 Go trials. Participants were instructed to focus on a small central fixation cross and press a button with their right hand as quickly as they could every time the Go cue appeared and to withhold their press when the NoGo cue appeared. Before the MEG recording all participants were given 40 practice trials, and confirmed that they had understood the task.

### EEG and MEG collection

MEG data were acquired continuously at 1 kHz in a magnetically-shielded room with a 306-channel Vectorview MEG system (Elekta Neuromag) that contained one magnetometer and two orthogonal planar gradiometers at each of 102 positions. EEG data were recorded simultaneously using a 70 electrode EEG ‘Easy Cap’, arranged according to the international 10–20 system. During recording, electrodes were referenced to the nose and the ground placed on the left cheek. Vertical and horizontal electrooculograms were recorded using paired EOG electrodes. Five head position indicator (HPI) coils were used to monitor head position. The 3D locations of the HPI coils, 80 ‘head points’ across the scalp, and three anatomical fiducials (the nasion and left and right pre-auricular points), were recorded using a 3D digitizer (Fastrak Polhemus Inc.).

Raw MEG data were initially preprocessed using MaxFilter software (version 2.0, Elekta-Neuromag) with movement compensation. Further preprocessing and data analysis of MEG and EEG used SPM12. Data were down sampled to 500 Hz, eye blink artefacts were corrected using the Berg method of artefact correction (a topography based artefact correction method, Berg and Scherg, 1994). Data were band pass filtered between 0.1 Hz and 40 Hz and divided into epochs of 900 ms (−100 ms before stimuli onset to 800 ms after) time locked to the stimulus onset, and baseline corrected (−100 to 0 ms). Epochs containing artefacts were rejected if the amplitudes exceeded the following thresholds: 2500 fT for magnetometers, 900 fT for gradiometers and 150 μV for the EEG. After artefact rejection the mean trial inclusions for the accurate Go and NoGo conditions for the control group was 383 [standard deviation (SD) = 44.2] and 94 (SD = 10); for the placebo session 346 (SD = 73.9) and 85 (SD = 17.9), and the citalopram session included 296 (SD = 109.3) and 77 (SD = 23.9), respectively. Robust averaging was used to average epochs for the successful Go trials and the successful NoGo trials.

### MRI

A magnetization prepared rapid acquisition gradient echo (MPRAGE) T_1_-weighted structural image was obtained from each subject (repetition time 2250 ms, echo time 2.99 ms, flip angle 9°, inversion time 900 ms, 256 × 256 × 192 isotropic 1 mm voxels) to co-register the MEG data and to enable subject specific modelling of the lead field for the source analysis.

The T_1_ images were also used in a voxel-based morphometry analysis to identify differences in grey matter volume between the bvFTD and controls groups. For this method, SPM 12 (www.fil.ion.ucl.ac.uk/spm) was used with the DARTEL toolbox (Ashburner, 2007). The T_1_ image of each subject was segmented into grey, white and CSF tissue classes and used together to create a study-specific group template, which improves the inter-subject alignment during normalization. The template was registered to MNI space, and used to generate Jacobian scaled modulated grey and white matter images from each subject, which were spatially normalized to MNI space and smoothed with an 8 mm full-width at half-maximum kernel. To examine differences between patients and controls, *t*-tests within SPM’s general linear model were performed on the grey and white images. Each model also included covariates of age and total intracranial volumes to correct for intersubject differences in global brain volume. Statistical images were thresholded with a cluster-based family-wise error (FWE) correction *P* < 0.05 (after *P* < 0.001 voxel-wise uncorrected threshold).

### Data analysis

Behavioural analyses examined mean reaction time for Go responses and incorrect NoGo responses and response accuracy (arsine transformed) using IBM SPSS Statistics 22.0®. Independent two sample *t*-tests were used to compare the reaction times of the 12 bvFTD patients on placebo and 20 controls, and Mann-Whitney U-tests were used for response accuracy (due to non-Guassian distribution). To compare the citalopram versus placebo sessions paired sample *t*-tests were used for reaction times and non-parametric Wilcoxon Signed Ranks test for response accuracy. Greenhouse-Geisser correction was used to correct for non-sphericity where necessary. Spearman’s rank correlation coefficient was calculated to estimate the relationship between NoGo accuracy and the Hayling scaled error score and the stereotypic and motor behaviours score of the Cambridge Behavioural Inventory. To investigate the relationship of NoGo disinhibition with general disinhibited behaviours, we also calculated a total ‘disinhibited/impulsive’ score from the Cambridge Behavioural Inventory, including the sum of all items from the disinhibited, challenging, motor, eating and insight subscales, and the euphoria items from the mood subscale. These specific types of behaviours have been shown previously to load onto a behavioural ‘disinhibition’ factor (Borroni *et al.*, 2012).

For the ERPs, peak amplitude and peak latency were measured from three midline electrodes Fz, Cz and Pz, for three ERP components: (i) the P2, the most positive peak after 150 ms maximal at Fz, included to examine early sensory processes preceding the N2; (ii) the N2, the most negative deflection after 200 ms maximal at Cz; and (iii) the P3, the most positive peak after 300 ms maximal at Pz. To test for disease effects, separate repeated measures ANOVAs were used to examine peak amplitudes and peak latencies. The three ERP components were included as the repeated measure (P2, N2 and P3) with subject group (bvFTD placebo versus controls) as the between subjects factor. To test the effects of citalopram treatment, a second set of repeated measures ANOVAs included the between subjects factors: ERP component (P2, N2 and P3) and drug (citalopram versus placebo), for the bvFTD patients who completed both sessions. As the peak amplitude for the N2 component is negative, absolute amplitude rather than signed amplitude was included in the analysis. Pairwise *t*-tests were Bonferroni corrected for multiple comparisons, and Greenhouse-Geisser corrected where appropriate.

Group differences between the NoGo source waveform contours were calculated time-point by time-point using two-sample *t*-tests between the bvFTD and control groups, and paired *t*-tests between the placebo and citalopram sessions across a 200 ms time window spanning the peak of each of the three ERP components of interest. Differences were considered significant if data points consecutively met the *P* < 0.05 criterion for at least 22 ms (11 data points with 500 Hz sampling) (*cf*. Guthrie and Buchwald, 1991).

Analyses of the MEG data used the 204 gradiometer MEG channels. Forward models were estimated using cortical meshes based on coregistering the fiducials and head shape points to the subject’s individual MRI scan. Inverse reconstruction was computed using the SPM12 ‘COH’ algorithm, comparable to standardized low-resolution brain electromagnetic tomography (sLORETA, Pascual-Marqui, 2002), that estimates distributed cortical responses across the entire brain volume. Images were computed for each subject for the successful Go and NoGo trials across the three time windows of interest spanning the three peak ERP components: the P2 (100 to 200 ms after stimuli onset), the N2 (250 to 350 ms) and the P3 (400 to 500 ms), and also baseline images for each trial type (−100 to 0 ms) to control for variance in between group comparisons.

A first set of general linear models included source images for controls and bvFTD placebo for the Go trials, NoGo trials and the baseline for each condition. Separate models were generated for each time window. Contrasts for the controls examined task performance (Go and NoGo trials versus baseline) and NoGo inhibition (NoGo versus Go trials). Contrasts of disease effects compared the bvFTD group with controls on the NoGo trials (the interaction term: Controls versus bvFTD × NoGo versus baseline).

A second set of general linear models tested the effects of citalopram in a within-subjects design. These models included images for citalopram and placebo sessions for the Go and NoGo trials and the baseline for each condition. Separate models were generated for each time window. Contrasts tested for drug effects on general task performance (Go and NoGo trials for citalopram versus placebo), and on NoGo inhibition (citalopram versus placebo × NoGo versus baseline).

Statistical maps of the normative data contrasts for the controls were thresholded with a cluster-based family-wise error correction *P* < 0.05 (after *P* < 0.001 voxel-wise uncorrected threshold). Contrasts between controls and bvFTD, and between the citalopram and placebo sessions used an independent regions of interest analysis. Two regions of interest were created using the Pickatlas ‘aal’ templates (Tzourio-Mazoyer *et al.*, 2002; Maldjian *et al.*, 2003, 2004) to examine right inferior frontal responses (including pars triangularis and pars opercularis) and right temporal involvement in response inhibition (masks are outlined in Fig. 3). These masks were used for small volume correction for the time windows of interest (*P* < 0.05 family-wise error corrected for multiple comparisons). In view of possible activation differences outside of our regions of interest, whole-brain analyses at the exploratory threshold of *P* < 0.001 (uncorrected) are also reported.

## Results

### Behavioural results

Mean reaction times and accuracy rates (arcsine transformed) for both Go and NoGo conditions are presented in Table 2. For all subjects, mean reaction times of the commission errors on NoGo trials were significantly faster than the reaction times for the Go trials [mean reaction times: Controls: Go trials = 287.60 ms, NoGo trials = 222.39 ms, *t*(18) = 5.7, *P* < 0.05; bvFTD placebo: Go trials = 475.98 ms NoGo trials = 369.04 ms, *t*(8) = 2, *P* < 0.05; bvFTD citalopram: Go trials = 538.81 ms NoGo trials = 332.03 ms, *t*(9) = 4, *P* < 0.05] demonstrating a response prepotency generated by the Go trials that must be inhibited on successful NoGo trials.

**Table 2:** Mean reaction times (in ms) and accuracy rates (arcsin transformed in radians, and non-transformed mean accuracy %) for Go (correct trials) and NoGo (commission errors) trials

	Between groups		Repeated measures	
	Controls (*n* = 20)	Placebo (*n* = 12)	Placebo (*n* = 10)	Citalopram (*n* = 10)
Reaction times (ms)				
Go	287.60 (9.58)	475.98 (42.04)	477.84 (46.62)	538.81 (53.33)
NoGo	222.39 (13.07)	369.04 (71.27)	314.64 (52.21)	332.03 (45.79)
Accuracy (rad)				
Go	1.5 (0.02)	1.4 (0.03)	1.4 (0.03)	1.34 (0.05)
NoGo	1.35 (0.03)	1.2 (0.13)	1.3 (0.12)	1.2 (0.14)
Accuracy (%)				
Go	99 (0.4)	96 (1.2)	96 (1.3)	93 (3.1)
NoGo	94 (1.3)	80 (10.0)	85 (8.9)	87 (10.0)

Standard errors in parentheses.

Compared to controls, the bvFTD placebo group were significantly slower when responding to the Go trials [*t*(30) = −5, *P* < 0.001] and they were slower when making commission errors on the NoGo trials [*t*(26) =2.8, *P* < 0.05]. The bvFTD placebo group also made significantly more omission errors on Go trials [mean Go accuracy: controls 99%, placebo 96%; U(30) = 20.5, *P* < 0.05], but at a group level they did not make significantly more commission errors on NoGo trials compared to controls [mean NoGo accuracy: controls 94%, placebo 80%; U(30) = 100, *P* ≥ 0.05].

Consistent with disinhibition underlying the commission errors, NoGo accuracy in the bvFTD placebo group correlated with the Hayling scaled error score (Spearman’s rho = −0.52, *P* < 0.05) and the stereotypic and motor behaviours score of the Cambridge Behavioural Inventory (Spearman’s rho = −0.68, *P* < 0.05), suggesting that patients with higher behavioural disinhibition on clinical measures were more likely to respond in error on a NoGo trial. The composite score of ‘general disinhibition’ calculated from the Cambridge Behavioural Inventory subscales (*cf*. Borroni *et al.* 2012), correlated with NoGo accuracy (Spearman’s rho = −0.57, *P* < 0.05), as did the total score of the Cambridge Behavioural Inventory (Spearman’s rho = −0.67, *P* < 0.05), suggesting that increased ‘everyday’ behavioural disinhibition is associated with more commission errors on the NoGo trials.

There were no significant within-group serotonergic effects on accuracy or reaction times in bvFTD (this is consistent with previous data in healthy subjects, *cf*. Macoveanu *et al.*, 2013). The patients’ behaviour may suggest a poor trade-off between the two conditions: the slow response and higher rate of omissions on the Go trials may facilitate inhibition on NoGo trials. However, there was no significant correlation between Go reaction times and NoGo accuracy (Spearman’s rho = 0.08 ns, bvFTD placebo, Spearman’s rho = 0.01 ns, bvFTD citalopram) suggesting that this was not the case.

Two patients performed poorly on the task, making a majority of NoGo commission errors (mean 92% NoGo errors). These two patients were excluded from further MEG and EEG analyses, which needed a sufficient number of trials to examine successful NoGo responses. This constraint goes beyond the general issue of ‘scanning patients with tasks they can perform’ (Price and Friston, 1999) and reflects the need for good signal-to-noise, which is influenced by the number of trials to obtain an accurate estimation of the magneto- and electro-physiological indices.

### Volumetric-based morphometry

**Figure 1:**
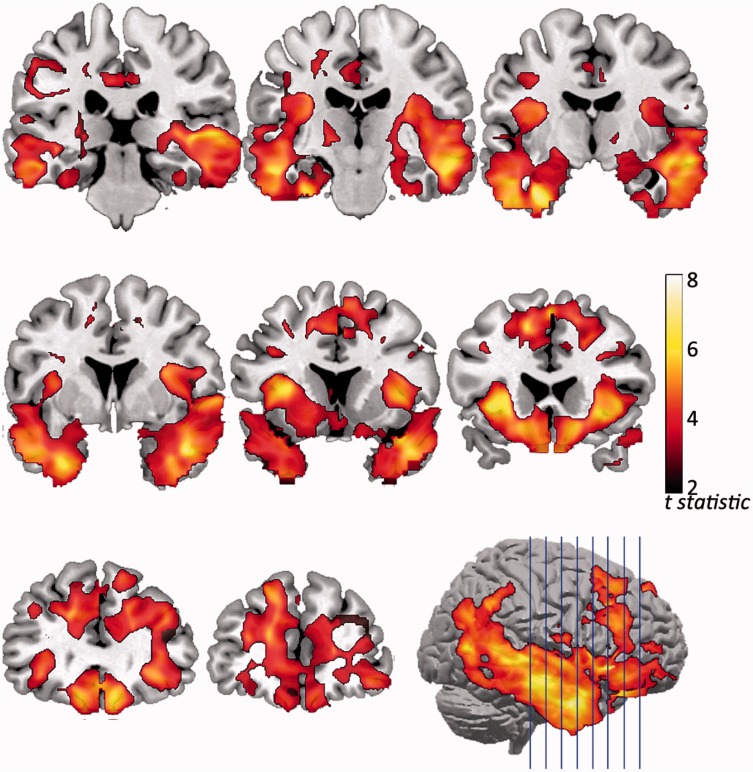
**The regions of grey matter loss in patients with bvFTD compared to the control group.** Atrophy is evident in inferior and middle temporal gyrus, inferior and superior frontal gyrus, bilateral temporal poles and orbitofrontal gyrus. Images are thresholded with a cluster-based family-wise error correction *P* < 0.05 (after *P* < 0.001 voxel-wise uncorrected threshold).

The voxel-based morphometry confirmed extensive grey matter atrophy for the patient group in the bilateral temporal poles, inferior and middle temporal gyrus, insula, inferior and superior frontal gyrus and orbitofrontal gyrus (Fig. 1). The atrophy measured by voxel-based morphometry is indicative of underlying pathology, although the presence of TARDBP (also known as TDP-43) or tau-positive inclusions cannot be measured or distinguished directly using voxel-based morphometry.

### EEG results

**Figure 2:**
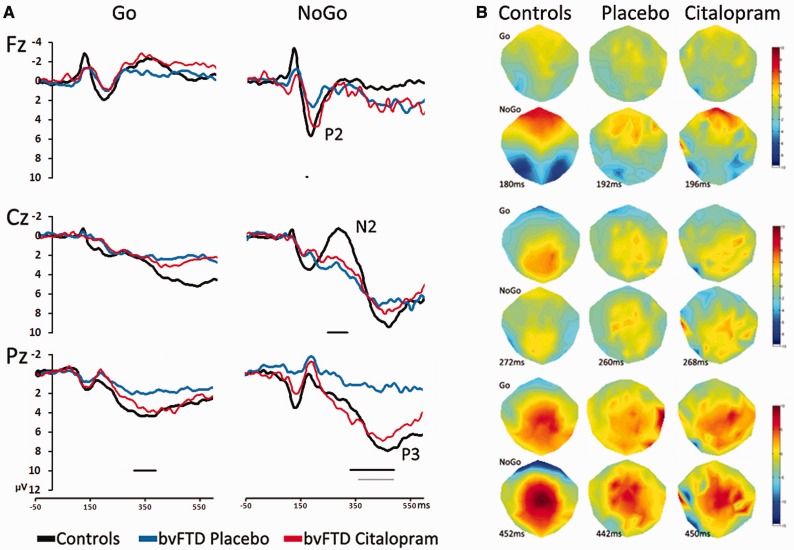
**Neurophysiological responses by task and drug conditions.** (**A**) ERPs from three midline electrodes, Fz, Cz and Pz for successful Go and NoGo trials. Time point 0 denotes the stimulus onset and for convention, negativity is plotted upwards. For NoGo trials, the bvFTD placebo group (*n* = 10) show significant reductions in peak amplitudes of all three components of interest: the P2 at Fz, N2 at Cz and P3 at Pz compared to controls. Citalopram enhanced the P3 at Pz in bvFTD (*n* = 9), restoring the amplitude towards normal levels compared to placebo. The black and grey horizontal lines indicate significant differences in onset latencies between controls and bvFTD on placebo, and citalopram versus placebo, respectively. (**B**) Topographies of the peak response for the P2, N2 and P3 components during the NoGo trials, with the Go trials presented at the same latency for comparison.

The analysis of the ERP data from the NoGo trials examined peak amplitude at three electrode sites (Fz, Cz, Pz) for the three components of interest (P2, N2, P3). Figure 2 depicts the mean ERP waveforms at each electrode site. In the bvFTD placebo group, peak amplitudes were diminished compared to controls, [*F*(1,24) = 27.05, *P* < 0.05], and pairwise *t*-tests revealed these differences as significant for the N2 at Cz (mean difference = 3.8 µV, SE = 1.7, *P* < 0.05) and P3 at Pz (mean difference = 6.4 µV, SE = 1.5, *P* < 0.001), but not the P2 at Fz (mean difference = 2.1 µV, SE = 1.4, *P* = not significant). There were no significant latency differences between the controls and placebo group, suggesting that although peak amplitudes are reduced, the peaks are not occurring significantly later in the patient group.

Comparisons of peak amplitudes between the placebo and citalopram sessions in bvFTD patients revealed a trend towards an overall effect of citalopram [*F*(1,8) =4.8, *P* = 0.059]. Pairwise *t*-tests revealed the P3 amplitude to be significantly enhanced during the citalopram session compared to placebo (mean difference = −4.9 µV, 1.8 SE *P* = 0.03]. The difference in P3 peak amplitudes between the placebo and citalopram sessions correlated with the Cambridge Behavioural Inventory stereotypic and motor behaviours subscale (Spearman’s rho = −0.54, *P* = 0.055, one-tailed), suggesting that the P3 was enhanced more in those patients who have fewer disinhibited behaviours. There were no group-wise differences in peak latency between citalopram and placebo sessions. Table 3 shows mean peak amplitude and peak latency for the controls and patients groups.

**Table 3:** Peak amplitude (µV) and latency (ms) for NoGo trials

	*Between groups*		*Repeated measures*	
	Controls (*n* = 20)	Placebo (*n* = 10)	Placebo (*n* = 9)	Citalopram (*n* = 9)
*P2 (Fz)*				
Peak Amp	6.2 (0.8)	4.0 (1.1)	4.1 (1.2)	6.0 (1.2)
Latency	180 (5.6)	193.4 (7.2)	194 (12.1)	196.7 (7.6)
*N2 (Cz)*				
Peak Amp	−2.1 (1.0)	1.6 (1.3)	1.2 (1.6)	1.4 (1.3)
Latency	272.5 (7.7)	260 (9.8)	257.8 (8.0)	269.3 (6.3)
*P3 (Pz)*				
Peak Amp	8.9 (0.9)	2.5 (1.2)	2.5 (0.9)	8.0 (1.9)
Latency	453.4 (7.9)	442.2 (10.0)	439.5 (9.0)	451.11 (11.4)

Standard error in parentheses.

The N2 at Cz for patients did not reach a negative threshold, but in all subjects is calculated as the most negative peak after 200 ms.

The potential differences between waveforms, measured using time-point by time-point *t*-tests, substantiate these results: the bvFTD placebo group compared to controls had a significant increase in onset latency of each of the three components of interest, reflected by reduced amplitudes in the rising flank of the deflections. For the NoGo-N2 at Cz and the NoGo-P3 at Pz the differences persisted, resulting in reduced amplitudes throughout the deflection. Comparing the citalopram and placebo sessions revealed a significant difference in the amplitudes of the NoGo-P3 at Pz, which was sustained after 250 ms. The significant point-by-point results are illustrated in Fig. 2.

### MEG results

**Figure 3:**
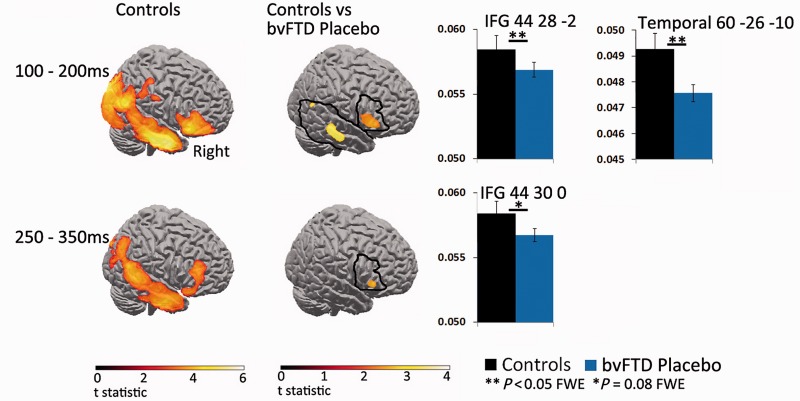
**sLORETA contrast images for successful NoGo trials, for controls and bvFTD patients on placebo.** Controls show sustained frontal and temporal source responses across the first two time windows (100–200 and 250–350 ms), which are greater for NoGo than Go trials (*P* < 0.05 FWE). For the bvFTD placebo group, compared to controls, right frontal and temporal sources are significantly reduced for NoGo trials from 100–200 ms (*P* < 0.05 FWE corrected within region of interest), and a trend for a reduced right inferior frontal gyrus response after 250 ms (*P* = 0.08 FWE corrected within region of interest). Data plots show peak differences in NoGo source responses between the control and placebo groups. The regions of interest of right inferior frontal gyrus and right temporal lobe are outlined in black.

During the first time window (100 to 200 ms, Fig. 3) for the control group, both Go and NoGo cues activated bilateral visual and prestriate cortex. There were greater source currents for NoGo versus Go trials in right temporal and right inferior frontal gyrus (*P* < 0.05, corrected). NoGo source activation was significantly reduced in the bvFTD placebo versus controls, within the region of interest mask of the right IFG and the right temporal lobe, (*P* < 0.05, corrected). Exploratory analyses at the liberal threshold *P* < 0.001 (uncorrected) revealed a single additional peak difference in the left occipital region between the controls and bvFTD placebo group. Citalopram did not change activation in the regions of interest in this early time window, and there were no significant peak differences at *P* < 0.001 uncorrected levels.

**Figure 4:**
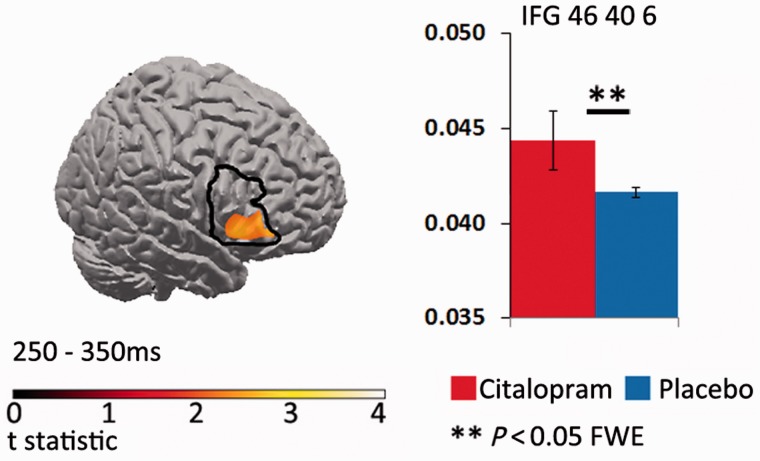
**sLORETA contrast of citalopram versus placebo for successful NoGo trials in patients with bvFTD, during the 250–350 ms time window.** Citalopram enhanced activation (mean current source density) in the right inferior frontal gyrus (*P* < 0.05 FWE corrected within region of interest). Data plot shows peak difference between citalopram and placebo within the right inferior frontal gyrus region of interest, for NoGo trials.

During the second time window (250 to 350 ms, Fig. 3), in the control group, NoGo compared to Go trials, revealed significant clusters in right temporal and right inferior frontal gyrus (*P* < 0.05, FWE corrected). There was a trend of reduced activation for NoGo trials in the right IFG for the bvFTD placebo compared to controls (*P* = 0.08, FWE corrected). Exploratory analyses (*P* < 0.001 uncorrected) revealed no additional peak differences between the controls and bvFTD placebo group. Importantly, in the citalopram session, right IFG activity was significantly increased in the right inferior frontal gyrus region of interest compared to the placebo session for successful NoGo trials (*P* < 0.05, FWE corrected, Fig. 4). The exploratory analyses revealed no additional peak differences between the citalopram and placebo sessions at *P* < 0.001 (uncorrected).

For the third time window (400 to 500 ms), in controls the MEG was not sensitive to cortical sources. There were no significant MEG-derived sources localized above *P* < 0.001 uncorrected. This is in contrast to the observation of a strong P3 in the EEG data within this time window. Potential reasons for the insensitivity of MEG to source generators of the P3 are discussed later, but this null result is consistent with the MEG literature on P3 responses.

## Discussion

The key results of this study demonstrate that in bvFTD, (i) patients could perform a simple NoGo task to some degree, but were impaired behaviourally; (ii) the neurophysiological markers of successful response inhibition were diminished, including the absence of the N2 evoked response, and reduced P3 evoked response, together with attenuated cortical sources in right temporal pole and right inferior frontal gyrus; and (iii) critically, citalopram restored the P3 and enhanced the cortical response of the right inferior frontal gyrus during NoGo inhibition.

These neurophysiological changes with disease and the favourable response to citalopram have important implications for understanding cognitive dysfunction in bvFTD, despite the fact that the neurophysiology appeared to be more sensitive to treatment than behavioural measures (no significant behavioural effects of citalopram were observed in this group of 12 participants). The results highlight the impaired neural substrates involved in response inhibition, including right prefrontal cortex, and the role of serotonin in regulating cognition. Further, they confirm that even atrophic and dysfunctional frontal cortical systems in bvFTD can be functionally restored to some extent by pharmacological manipulation.

The right inferior frontal gyrus has been specifically identified as an important locus of control during response inhibition (Aron *et al.*, 2004, 2014; Konishi *et al.*, 1998, 1999) and in the healthy control subjects a sustained response in this region was evident during the NoGo trials. The attenuation of the response in inferior frontal gyrus in bvFTD is commensurate with reports that lesions to this region impair inhibition (Aron *et al.*, 2003; Kopp *et al.*, 2013). The focal enhanced response in the inferior frontal gyrus to citalopram suggests that, although this region is part of the dysfunctional frontotemporal network, its neuronal responses can be modulated by serotonergic manipulation. This is consistent with changes in activation of inferior frontal gyrus after serotonergic modulation in health (Macoveanu *et al.*, 2013) and in Parkinson’s disease (Ye *et al.*, 2014). Parkinson’s disease, like bvFTD, is characterized by impulsivity with poor response inhibition (Weintraub *et al.*, 2010; Bentivoglio *et al.*, 2012). The problem of impulsivity in Parkinson’s disease is not restricted to patients with impulse control disorders: patients who do not have impulse control disorders are still impulsive on a wide range of measures (Nombela *et al.*, 2014; *cf*. Jahanshahi *et al.*, 2015) and this impulsivity has also been linked to serotonergic depletion (Politis *et al.*, 2010, 2012). Interestingly, despite clear differences between the clinical syndromes of Parkinson’s disease and bvFTD, both disorders have dysfunctional frontal cortico-striatal pathways which may contribute to impulsivity (O'Callaghan *et al.*, 2013*b*). The response in both groups to citalopram underlines the importance of serotonergic transmission in frontostriatal circuits for response inhibition.

Despite the enhanced response of the inferior frontal gyrus with citalopram in bvFTD, there was no concomitant improvement in the NoGo-N2 component of the EEG evoked potential, which peaks within the same time window. Indeed, the NoGo-N2 was absent in bvFTD in both sessions but the NoGo-P3 was significantly enhanced by citalopram, indicating differing sensitivities of these components to serotonergic modulation. Corroborative effects of serotonergic transmission in health on the NoGo-P3, but not NoGo-N2, have been reported: subjects without the −1019G allele who have putatively elevated serotonergic levels (Beste *et al.*, 2011) and subjects with high trait impulsivity (Ruchsow *et al.*, 2008) make more NoGo commission errors and have a reduced NoGo-P3 to unpredictable NoGo trials. However, the effects of serotonin in health appear initially to contrast with our results in bvFTD, in whom increasing serotonin enhanced the P3 and inferior frontal gyrus response. For example, in healthy individuals with fewer 5HT2A receptors, reducing serotonin availability via acute tryptophan depletion increased the inferior frontal gyrus response during NoGo trials (Macoveanu *et al.*, 2013) whereas in our study, increasing serotonin neurotransmission in the patient group (who likely have fewer 5HT2A receptors) (Bowen *et al.*, 2008), enhanced the response in inferior frontal gyrus. In addition, FTD patients with the long polymorphism of the 5-HTTLPR (*SLC6A4*) gene, had a better cognitive profile relative to cortical damage compared to patients with the short allele (Premi *et al.*, 2015). Together, these studies suggest that there is an optimal degree of serotonergic transmission for cognition and behavioural control. Excessive levels of serotonin in health or deficiencies in disease both impair performance (*cf*. Deakin *et al.*, 2004). This effect is analogous to the well-established ‘inverted U-shaped’ function relating dopaminergic transmission to cognitive and motor control and regional neuronal activation (Rowe *et al.*, 2008; Cools and D'Esposito, 2011). The corollary is that citalopram treatment would only be expected to improve the neural systems for inhibitory control if a subject was in a relatively hypo-serotonergic state such as bvFTD.

In bvFTD, the reduced amplitudes of the EEG evoked response and decreased MEG signal in inferior frontal and temporal cortex reflects the loss of specific neurocognitive functions relevant to NoGo inhibition. Each ERP component is considered a separate measure of a multifaceted neurocognitive network involved in inhibition. The N2 is suggested to represent conflict monitoring, because it is present for infrequent Go as well as NoGo trials (Nieuwenhuis *et al.*, 2003; Donkers and van Boxtel, 2004; Smith *et al.*, 2010; Randall and Smith, 2011). In contrast, the NoGo-P3 is evident when the inhibition of planned responses is required (Randall and Smith, 2011), and is thus considered an index of motor inhibition (Smith *et al.*, 2008, 2013; Enriquez-Geppert *et al.*, 2010), and also the evaluation of the inhibitory process (Roche *et al.*, 2005). The observed reductions in both the ERP components in bvFTD may reflect impaired detection of behaviourally salient low frequency events and impaired motor inhibition. Critically, serotonin may be only effective in improving the cognitive processes underlying the P3, consistent with evidence that serotonin manipulations affect the response inhibition component of NoGo trials (Macoveanu *et al.*, 2013), and mediate performance monitoring during the Go-NoGo task (Evers *et al.*, 2006).

Despite the improvements in neuronal response in the inferior frontal gyrus and P3 with citalopram, we did not observe group-level changes in behaviour: patients’ accuracy and reaction times did not significantly improve across sessions. It could be considered that NoGo accuracy might be due to lack of visual focus or attention to the task, because patients’ Go trial accuracy is also reduced compared to controls. However the proportion of Go errors (mean 4%) and NoGo accuracy (mean 80%) in the placebo group does not support this alternative interpretation. Moreover, the evidence of faster responses when making a NoGo commission error compared to the reaction time of Go trials, suggests that the Go trials are inducing a prepotent response that must be inhibited on NoGo trials. Comparably, not all studies of serotonergic interventions find significant behavioural effects, even in the presence of neuronal changes (Rubia *et al.*, 2005; Evers *et al.*, 2006; Macoveanu *et al.*, 2013). However, some earlier open label studies of SSRI’s in bvFTD have shown significant improvements in behaviour (Boxer and Boeve, 2007). Behavioural improvement may also be limited without additional restoration of the neuronal connectivity of the inferior frontal gyrus, or sources underlying the NoGo-N2. This raises the question of whether enhancing additional neuronal regions would be necessary, and sufficient, to improve disinhibition in bvFTD. The patients with bvFTD also had impaired temporal cortical responses, compared to controls, which were not enhanced by citalopram. It may also be the case that marked individual differences in the therapeutic response mask a group effect (*cf*. Ye *et al.*, 2014). Ye *et al.* revealed that clinical and demographic variables of the patients, together with measures of the integrity of structural frontostriatal networks, determined the response to citalopram. A larger sample of patients with bvFTD, in combination with genotyping and structural analyses, may enable stratification of patients to examine who is most likely to benefit from SSRIs at a single-subject behavioural level.

There are important pharmacological issues to consider when using SSRIs. For example, it has been questioned whether chronic SSRI administration (>2 weeks) has the same effect as a single dose. Research on fear responses has suggested differential effects of acute, subchronic or chronic dosing of SSRI’s in human (*cf*. Harmer and Cowen, 2013) and animal models (Burghardt *et al.*, 2004). However, one should be cautious not to extrapolate from these effects on affective responses to cognitive and motor tasks, since serotonin has separable effects on the neuronal substrates of each network (Del-Ben *et al.*, 2005; Cools *et al.*, 2011). For example, for prefrontal systems mediating reversal learning or motor response inhibition, there are similar effects of acute and chronic administration (Almeida *et al.*, 2010; Bari *et al.*, 2010). Moreover, low dose SSRIs may induce 5HT1A inhibitory autoreceptors leading to a reduction in neurotransmission, whereas higher doses induce a net increase in neurotransmission (Bari *et al.*, 2010). At the dose of citalopram we used, and for the NoGo inhibition task dependent on prefrontal cortex, the evidence suggests a net increase in neurotransmission. Although this is consistent with the behavioural benefit on motor response inhibition in Parkinson’s disease (Ye *et al.*2014), the clinical benefit of a SSRI for bvFTD disinhibition remains to be shown in clinical trials with chronic treatment. We suggest that the serotonergic modulation of frontal inhibitory systems as revealed by MEG in this study is relevant to behaviour, and validates a translational pathway from animal and pharmacological models towards future larger trials with clinical endpoints.

One methodological limitation was the source localization from the MEG data which were restricted to the early time windows, up to 350 ms. Additional cortical regions that generated the P3 component were not identified, possibly because the MEG source localization during this time window is poor. For example, MEG is relatively insensitive to neuronal sources in gyral convexities because of their radial orientation, while EEG is more sensitive to these sources. This may explain in part why the P3 was not evident in the MEG but seen clearly in the EEG recording. The localization of the P3 time window using EEG data has identified bilateral premotor and left inferior frontal regions (Albert *et al.*, 2013), which accords with results from functional MRI (Smith *et al.*2013). It should be noted however, that both EEG and MEG measures are relatively insensitive to deeper sources, such as those generated in the basal ganglia. Thus it is difficult to observe direct pharmacological effects in the striatum. Alternative methods may be more appropriate for examining contributions of serotonergic manipulation to frontostriatal interactions. Given the sensitivity of MEG to depth, one must consider the impact of atrophy on signal-to-noise in clinical populations, which may increase the distance from cortex to sensors by several millimetres. Atrophy and greater cortical depth in bvFTD cannot explain our most important result, that citalopram enhances EEG components and MEG source current density, as cortical depth would be unchanged by acute drug treatment.

Finally, one must consider the heterogeneity of the patient population. While disinhibition is a criterion for the diagnosis, it is not obligatory, and the extent of inhibition varies considerably between patients and over the course of the illness. Although we did not select patients according to their cognitive profile within bvFTD, we cannot rule out biases resulting from an interaction between impulsivity and referral or consent to participate. Nonetheless, our patients manifested typical disinhibition, both on the NoGo task and neuropsychological tests such as the Hayling Test, as well as endorsements of symptoms of behavioural disinhibition from relatives or carers on the Cambridge Behavioural Inventory, comparable to previous research (O'Callaghan *et al.*, 2013*b*).

In conclusion, we have shown that bvFTD impairs the neurophysiological signature of critical regions for response inhibition, and that the response of the right inferior frontal gyrus can be partially reversed by citalopram. Optimization of serotonergic strategies to treat disinhibition and other aspects of impulsivity will require clinical trials, and the identification of factors that determine individual differences in therapeutic efficacy.

## Funding

This work was primarily funded by the Wellcome Trust (088324, 103838 to J.B.R.) with additional support from the Medical Research Council (MC US A060 0016, and RG62761) and the National Institute for Health Research’s Cambridge Biomedical Research Centre. The BCNI is supported by a joint award from the Wellcome Trust and Medical Research Council.

## Abbreviations

bvFTD: behavioural variant of frontotemporal dementia
ERP: event-related potential
SSRI: selective serotonin reuptake inhibitor
